# Comparison of gross tumor volumes of pulmonary metastasis defined by CT and MRI in 0.345 T MRI-guided radiotherapy

**DOI:** 10.1259/bjro.20200010

**Published:** 2020-07-31

**Authors:** Yukihiro Hama, Etsuko Tate

**Affiliations:** 1 Department of Radiation Oncology, Tokyo-Edogawa Cancer Centre, Edogawa Hospital, Tokyo, Japan

## Abstract

**Objective:**

To assess the difference in gross tumor volumes (GTVs) defined by CT (GTV-CT) and by low magnetic field strength (0.345 T) MRI (GTV-MRI) in patients simulated for MRI-guided radiotherapy forlung metastasis.

**Methods:**

28 patients (148 lesions) who underwent CT and MRI simulation with the tri-60Co MRI-guided radiotherapy system (MRIdian, ViewRay) were included in this study. GTV-CT and GTV-MRI were compared using the paired *t*-test. The equivalence of variance between GTV-CT and GTV-MRI of small lesions (GTV-CT <1 ml) and large ones (GTV-CT >= 1 ml) was evaluated using F-test. The correlation between GTV-CT and GTV-MRI was evaluated by the correlation coefficient.

**Results:**

GTV-MRI was 120% larger than GTV-CT (*p* < 0.001) for small lesions, whereas GTV-MRI was 40% larger than GTV-CT (*p* < 0.001) for large lesions. In small lesions, the variation in GTV-MRI was significantly larger than that of GTV-CT (*p* < 0.001). There was no significant difference in the variation of GTV-MRI and GTV-CT in large lesions (*p* = 0.121). The correlation coefficient for small lesions was 0.93, whereas that for large lesions was 0.99, with large lesions having better correlation.

**Conclusions:**

GTV-MRI was larger than GTV-CT and the correlation between GTV-MRI and GTV-CT was better in large lesions. If the tumor volume is 1 ml or larger, the lesion can be accurately monitored even with a low magnetic field strength MRI.

**Advances in knowledge:**

This study is the first clinical report to evaluate the tolerability of MRI images in 0.345 T MRI-guided radiotherapy for lung metastasis. GTV contoured by MRI was larger than GTV by CT, and this tendency was more pronounced in small tumors of less than 1 ml.

## Introduction

MRI-guided radiation therapy provides a real-time view of the tumor and surrounding normal tissue, allowing treatment with a minimum margin setting for the moving target.^1,2^ Since CT has better spatial resolution of lung tumors than MRI, it is important to know the limitations of MRI when performing radiotherapy for lung tumors under MRI guidance. Recently, it has been reported that the interobserver variability is larger for MRI-based gross target volume (GTV) delineation compared to that based on CT in patients simulated for radiotherapy for lung tumors.^3–5^ However, it has not been investigated whether the GTV based on MRI (GTV-MRI) is larger or smaller than that on CT (GTV-CT), or whether there is a certain trend between GTV-CT and GTV-MRI among patients with lung metastasis.

The purpose of this study is to investigate whether there is a difference between GTV-CT and GTV-MRI, and if there is a difference, verify how they differ.

## Methods and materials

All procedures performed in this study were in accordance with the ethical standards of the institutional and/or national research committee and with the 1964 Helsinki Declaration and its later amendments or comparable ethical standards. This prospective study was approved by the institutional review board (Approval number RO201800802), and written informed consent was obtained from the patients before treatment.

### Patients

28 consecutive patients with lung metastasis (148 lesions) who underwent both CT and MRI simulation with the tri-60Co MRI-guided radiotherapy system (MRIdian, ViewRay, OH) during the last 6 months were included in this study. There were 14 males and 14 females and the median patient age was 59.0 years (range 24–85 years, standard deviation [SD] 13.7 years).The primary sites were lung (*n* = 6,21.4%), colon (*n* = 5, 17.9%), rectum (*n* = 2, 7.1%), breast (*n* = 2, 7.1%), liver (*n* = 2, 7.1%), pancreas (*n* = 2, 7.1%), others (*n* = 9, 32.3%). In order to ensure the reproducibility of the position, a rigid and secure support around the patient was created by Vac-Lok^™^ Cushions and CT and MRI were taken in a supine position. In this study, not all patients who performed an MRI simulation received MRI-guided radiotherapy. After MRI simulation, patients who did not actually receive MRI-guided radiotherapy (*n* = 8/27) are also included.

### CT protocol

A breath-hold CT scan was performed with patients immobilized using a Vac-Lok^™^ Cushion in supine with their arms overhead. CT images were acquired during the end-expiratory phase with the breathing command. Multidetector CT was performed on a 64-slice GE Discovery CT 750HD scanner (GE Healthcare, Waukesha) with a slice thickness of 2.5 mm with no interslice gap.No intravenous contrast is used for these CT scans, and the whole procedure generally took about 20 min.

### MRI protocol

MRI was performed using an imaging component of the ViewRay systemwith patientsimmobilized using a Vac-Lok^™^ Cushion in supine with their arms overhead. The imaging component consists of split superconductor magnet (0.345 T) and the bore size is 70 cm with a maximum field of view of 500 × 450 mm (pixel size 0.668 × 0.668 mm). Imaging isocenter is coincident with the isocenter of radiotherapy component. The combination of spine and body array coils was used as the receiver. Patients were positioned head first and all imaging was performed at the magnet isocenter. Chest MRI was performed in the axial planes with use of a breath-hold, 3D, true fast imaging with steady-state precession (true-FISP) pulse sequence (repetition time [TR], 3.84 ms; echo time [TE], 1.92 ms; flip angle, 60). The slice thickness (Z-axis resolution) was 3 mm and spatial resolution in the longitudinal direction is 3 mm and the pixel size was 1 mm.MRI scans were acquired during the end-expiratory phase with the breathing command.

### Measurement of GTV

**Figure 1.**
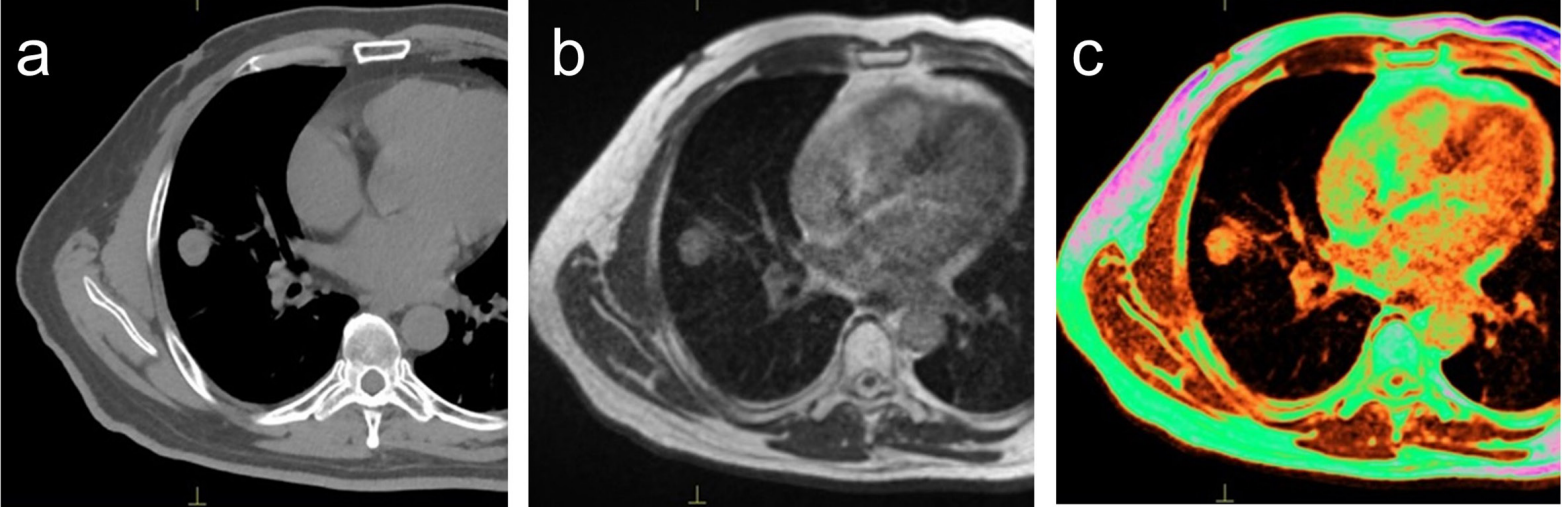
Measurement of GTV. A rigid fusion of both MRI (**a**) and CT (**b**) was performed prior to contouring. GTV based on CT (GTV-CT) was defined on the planning CT with a window level of 30–50 HU and window width of 350–500 HU. GTV based on MRI (GTV-MRI) was defined using fusion image (**c**) in order to avoid mistakenly containing normal tissues such as blood vessels. GTV, gross target volume.

Both GTV-MRI and GTV-CT were measured separately by two radiologists (ET, BS) using a commercial treatment planning software (Monaco^®^, Elekta, Stockholm, Sweden), and consensus was obtained. We performed target delineation using the threshold function installed in the software. Both GTV-CT and GTV-MRI were created by setting so that the portion where the signal intensity changes from 30 to 50% is recognized as the boundary line. A rigid fusion of both CT and MRI was performed prior to the calculation by the Monaco software (Figure 1a–c.). GTV-CT was defined on the planning CT with a window level of 30–50 HU and window width of 350–500 HU, and then GTV-MRI was defined using fusion images in order to avoid mistakenly containing normal tissues such as blood vessels. Selection criteria for metastatic lesions were foci detected in two or more slices by MRI. If multiple metastases were detected in the same lung segment, only the largest lesions were examined.

### Statistical analysis

We measured GTV-CT and GTV-MRI using radiotherapy treatment planning software (Monaco^®^, Elekta, Stockholm, Sweden) and compared them in two groups, small GTV (Group A: GTV-CT <1 ml) and large GTV (Group B: GTV-CT = or>1 ml).

The rationale to differentiate volume by 1 ml is as follows: the slice thickness at 0.345 T MRI is 3 mm, and if no mass is confirmed in two or more consecutive images, it will not be diagnosed as metastatic lung cancer. In order to obtain a cross-sectional image without partial volume effect in two or more consecutive slice sections, assuming a spherical tumor, the tumor must be at least 6 mm in the cranial direction and 6 mm in the caudal direction. For a spherical tumor with a radius of 6 mm, cross-sectional images can be obtained at any slice position over two consecutive slices. Since the volume of a sphere having a radius of 6 mm is about 1 ml, the volume of the cut-off was set to 1 ml.

GTV-CT and GTV-MRI were compared using the paired *t*-test. The coefficient of variation (CV) values of GTV-CT and GTV-MRI were calculated and the equivalence of variance between GTV-CT and GTV-MRI of Group A or Group B was evaluated using F-test. CV is a measure of relative variability or the ratio of the standard deviation (SD) to the mean (CV = SD/mean * 100). The correlation between GTV-CT and GTV-MRI was evaluated by the correlation coefficient (r). CV values of GTV-CT and GTV-MRI were compared using the paired t-test. *P* values of less than 0.05 were regarded as statistically significant.

## Results

**Figure 2.**
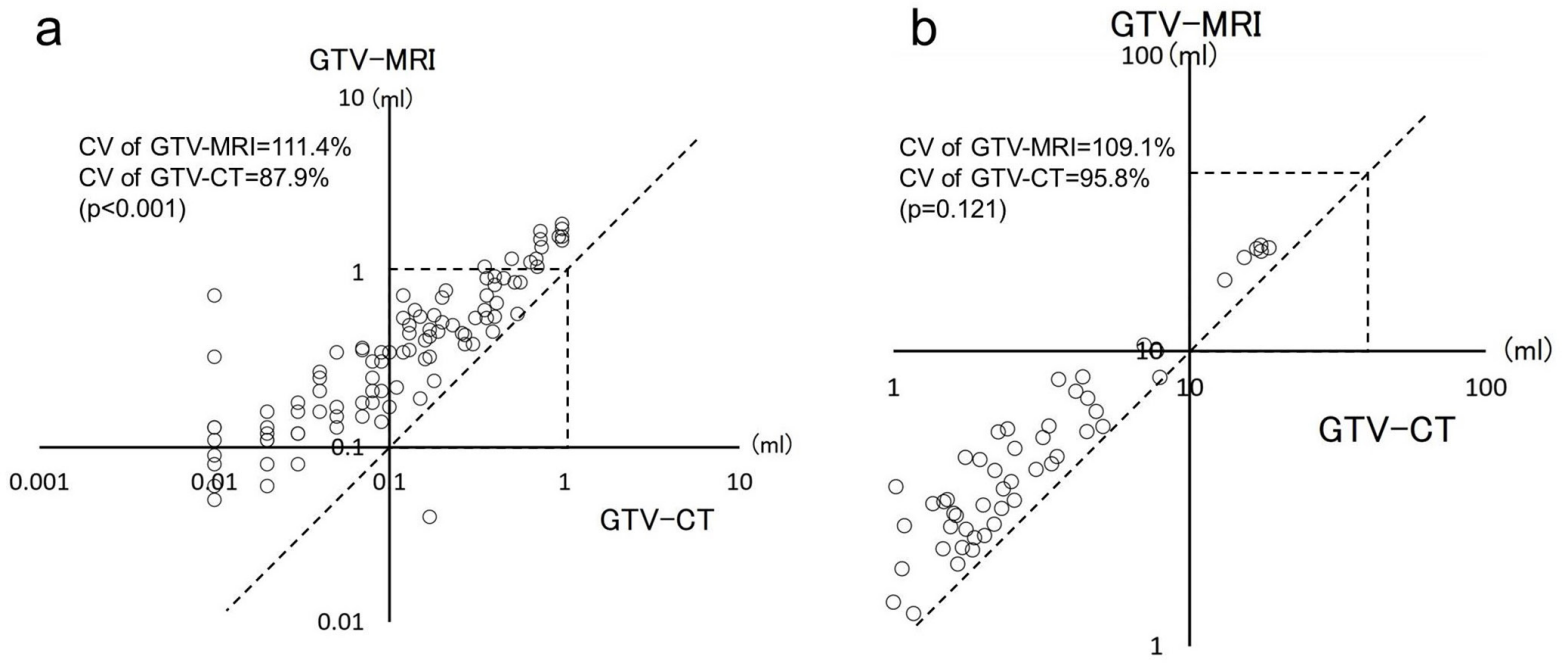
Relative variability of GTV-CT and GTV-MRI in small lesions (Group A) and large lesions (Group B). (**a**) Scatter plot of Group A. The coefficient of variation (CV) value of GTV-MRI was significantly larger than that of GTV-CT (111.4% *vs* 87.9%, *p* < 0.001). (**b**) Scatter plot of Group B.The CVvalue of GTV-MRI was larger than that ofGTV-CT, but there was no statistically significant difference(109.1% *vs* 95.8%, *p* = 0.121).The closer the plotted points are to the dotted line (*Y* = X), the higher the homology between GTV-MRI and GTV-CT. V, coefficient of variation; GTV, gross target volume.

In Group A, GTV-CT was 0.23 ± 0.26 (mean ± SD) ml and GTV-MRI was 0.51 ± 0.45 (mean ± SD) ml. GTV-MRI was found to be 120% greater than GTV-CT (*p* < 0.001). On the other hand, GTV-CT in Group B was 4.37 ± 4.77 (mean ± SD) ml and GTV-MRI was 6.21 ± 5.95 (mean ± SD) ml. GTV-MRI in Group B was found to be 40% greater than GTV-CT (*p* < 0.001). In Group A, CV of GTV-MRI was significantly larger than that of GTV-CT (111.4% *vs* 87.9%, *p* < 0.001) (Figure 2a). On the other hand, in Group B, CV of GTV-MRI was larger than that of GTV-CT, but there was no statistically significant difference (109.1% *vs* 95.8%, *p* = 0.121) (Figure 2b). The correlation coefficient (r) for small lesions in Group A was 0.93, but that for large lesions in Group B was 0.99, with large lesions having better correlation.

## Discussion

If the patient breathes, the tumor location may shift 5–20 mm^6,7^. MRI-guided radiation therapy system allows real-time monitoring of tumor and normal tissue locations during radiotherapy. MRI is better at displaying soft tissue than X-rays and other images, minimizing the risk of irradiating surrounding normal tissues. Because the risk of normal tissue damage is reduced, treatment is safer even with the same radiation dose.^8^ However, since the spatial resolution of MRI is inferior to that of CT, it is necessary to obtain accurate information regarding the reliability of MRI when setting a margin.

So far, there has been a report that evaluated the variability of GTV in MRI, where it deals with primary and metastatic lung cancer as the same population,^4^ the size of the tumor is larger than ours, it is insufficient for the study which evaluated the limitation of MRI. Our study is different from the previous study in that only metastatic lung cancer was analyzed, and the analysis was divided into tumors smaller than 1 ml and 1 ml or larger tumors. Metastatic lung cancer generally has no spicule or atelectasis and has a smooth border. We believe that analyzing the same type of tumor will allow us to accurately assess the limitations of lower field strength MRI. The results of the study show that the partial volume effect of the tumor is not deficient in the marginal signal, but is depicted as a positive signal. Based on the results of this study, it is suggested that PTV can be created without a margin on GTV-MRI.

This study has some limitations. First, MRI pulse sequences are not optimized to depict lung metastasis. Using volumetric zero echo time (ZTE) sequence, high-resolution structural information of small lung metastasis may be obtained.^9^ However, ZTE is not available on current MRI component of ViewRay system. Second, this is a retrospective study of a single-institutional experience. A multi-institutional prospective study of MRI-guided radiotherapy for patients with lung metastasis is desirable.

In conclusion, GTV-MRI was shown to be larger than GTV-CT, and the correlation between GTV-CT and GTV-MRI was better in large lesions. When performing MRI-guided radiotherapy for metastatic lung cancer, it is considered that the lesion can be accurately monitored even with a low magnetic field strength of 0.345 T if the tumor volume is 1 ml or larger.
